# Urinary leakage during sexual intercourse among women with incontinence: Incidence and risk factors

**DOI:** 10.1371/journal.pone.0177075

**Published:** 2017-05-24

**Authors:** Hui-Hsuan Lau, Wen-Chu Huang, Tsung-Hsien Su

**Affiliations:** 1 Department of Medicine, Mackay Medical College; Department of Obstetrics and Gynecology, Mackay Memorial Hospital; Mackay Medicine, Nursing and Management College, Taipei, Taiwan; 2 Department of Obstetrics and Gynecology, Hsinchu Mackay Memorial Hospital, Hsinchu, Taiwan; H Lee Moffitt Cancer Center and Research Institute, UNITED STATES

## Abstract

**Background:**

Coital incontinence is an under-reported disorder among women with urinary incontinence. Women seldom voluntarily report this condition, and as such, related data remains limited and is at times conflicting.

**Aims and objectives:**

To investigate the incidence and quality of life in women with coital incontinence and to determine associated predictors.

**Methods:**

This observational study involved 505 sexually active women attending the urogynecologic clinic for symptomatic urinary incontinence at a tertiary medical center. All of the patients were consulted about the experience of coital incontinence and completed evaluations including urodynamics, and valid questionnaires including the short form of the Pelvic Organ Prolapse/Urinary Incontinence Sexual Questionnaire, the Urogenital Distress Inventory and the Incontinence Impact Questionnaire.

**Results:**

Of these women, 281 (56%) had coital incontinence, while 224 (44%) did not. Among women with coital incontinence, 181 (64%) had urodynamic-proven stress incontinence, 29 (10%) had mixed incontinence, and 15 (5%) had detrusor overactivity. Only 25 (9%) sought consultation for this disorder before direct questioning. Fifty percent (84/281) of the women rarely or sometimes had incontinence during coitus, while 33% (92/281) often had incontinence, and 17% (48/281) always had incontinence. The frequency of coital incontinence was not different regarding the types of incontinence (p = 0.153). Women with mixed incontinence had the worst sexual quality of life and incontinence-related symptom distress. Based on univariate analysis, higher body mass index (OR 2.47, p = 0.027), and lower maximal urethral closure pressure (≤ 30 cmH2O) (OR 4.56, p = 0.007) were possible predictors for coital incontinence. Multivariate analysis showed lower MUCP was independently significant predictors (OR3.93, p = 0.042)

**Conclusions:**

The prevalence of coital intercourse in urinary incontinence women was high. Coital incontinence in these women was associated with abnormal urodynamic diagnosis and urethral dysfunction.

## Introduction

Coital incontinence is defined as “complaint of involuntary loss of urine during coitus” according to the International Urogynecological Association and the International Continence Society in 2010 [1]. Coital incontinence is a common but under-reported symptom that adversely affects sexually-active women. A literature review by Serati et al. searched related articles from 1970 to 2008 and reported the incidence of coital incontinence ranged between 10–27% [2]. However, two recent studies reported a higher prevalence of coital incontinence as up to 60% [3] and 67% [4]. Although urinary incontinence during coitus may be an embarrassing problem that may lead to reduced sexual desire, reduced ability to achieve an orgasm, and may even be harmful to a relationship, this issue is difficult to understand and research [5]. One reason may be that it would appear that women very seldom voluntarily consult on the issue of coital incontinence unless they are asked directly by physicians or asked to complete related questionnaires [6].

Due to the limited data on coital incontinence, the pathophysiology is yet to be well known. Aside from an unknown pathogenesis, its frequency and impact on quality of life are also unclear. According to the clinical findings of Hilton et al. in 1988, coital incontinence during penetration is more prevalent in women with stress incontinence, while incontinence at orgasm is more common in women with detrusor overactivity [7]. Thus, coital incontinence is generally divided into two forms: incontinence during penetration and incontinence at orgasm. However, Moran et al. in 1999 investigated 228 women with coital incontinence either during penetration or at orgasm. They reported that coital incontinence was more prevalent in patients with urodynamic stress incontinence, and not common in detrusor over-activity [6]. Among their patients, 80% had incontinence during penetration, 93% had incontinence at orgasm, and 92% had incontinence for both, indicating coital incontinence as a common symptom during sexual activity in women with stress incontinence. Thus, Moran et al. proposed urethral dysfunction as the possible causative of coital incontinence [6].

The pathophysiology of urinary incontinence has not yet been fully understood [4]. As a result, further investigation into this issue is warranted [8]. The present study aimed to evaluate the incidence, frequency, and risk factors of coital incontinence among women with incontinence. Urethral function and sexual quality of life of those with coital incontinence were also investigated.

## Materials and methods

All sexually active women with urinary incontinence attending the out-patient Urologic Clinic of a tertiary medical center were recruited using convenience sampling, and consecutively interviewed about their experience with regards to coital incontinence from April 2014 to March 2015. The clinical evaluation included medical history, physical examination, and urine analysis. Face-to-face interviews were conducted by a research nurse in a quite conference room beside the clinic. The women were asked questions regarding their experiences with regards to urinary incontinence during intercourse (either urine leakage during penetration or at orgasm). The frequency of coital incontinence was evaluated by 5-point Likert scale (Never, Rarely, Sometimes, Often, and Always). Women who never had coital incontinence were recruited as a comparison group during the interview. Patients underwent urodynamic measurements, pelvic examination for staging of prolapse according to the pelvic organ prolapse quantification (POP-Q) system [9] and valid questionnaires to evaluate their quality of life, including the short form of the Pelvic Organ Prolapse/Urinary Incontinence Sexual Questionnaire (PISQ-12) [10], the Urogenital Distress Inventory (UDI-6) and the Incontinence Impact Questionnaire (IIQ-7) [11]. Patients were excluded if they did not complete all of the evaluations or if they had urinary tract infection, any major medical condition (any chronic illness, such as poor-controlled diabetes, cardio-vascular disease, cancer, end-stage renal failure, or neurological disease), having stage 2 or more prolapse, or psychiatric disease that might influence the urodynamic measurements or questionnaire scoring. All participants provided verbal informed consent to participate in this study. Verbal consent contained all elements of this study, and the participant verbally agreed to participate. The Institutional Research Board of Mackay Memorial Hospital approved this study. The approval number is 15MMHIS080e.

PISQ-12 which was used to assess sexual function in women with pelvic organ prolapse and/or incontinence included 3 domains: behavioral-emotive (items 1–4), physical (items 5–9) and partner-related (items 10–12). The response of each item was scored from 0 to 4, with a total score of 0–48. A higher score indicated better sexual function [10]. The UDI-6 and IIQ-7 were designed to assess symptoms and quality of life related to urinary incontinence. UDI-6 was composed of 6 items including 3 subscales: irritative, discomfort/obstructive, and stress symptoms. Each item was scored from 0 to 4. IIQ-7 which was designed to evaluate incontinence-related quality life impairment was composed of 7 items, and included 4 domains: relationships, travel, emotional health, and physical activity. Each item was also scored from 0–4. For UDI-6 and IIQ-7, a higher score indicated worse symptoms and quality of life [11]. Urodynamic studies (UD 2000, Medical Measurement System, Enschede, Netherlands) included spontaneous uro-flowmetry, filling and voiding cystometry, and urethral pressure profile study. All urodynamic assessments were performed using standard procedures as described previously [12,13]. The presence of urodynamic stress incontinence (USI) and detrusor over-activity (DO) were recorded. The terminology used in this paper conformed to the standardization of terminology for female pelvic floor disorders from the International Urogynecological Association/ International Continence Society joint report [1].

Statistical analysis was performed using one-way analysis of variance (ANOVA), Mann-Whitney U test, or independent t-test for continuous variables, and the chi-square or Fisher’s exact test for categorical variables, as appropriate. Univariate analysis was used to assess the association of potential predictive factors of coital incontinence and significant variables were entered into a multivariate analysis that was performed using logistic regression. Statistical analysis was performed using the Statistical Package for the Social Sciences (SPSS) 17.0 for Window (SSPS, Chicago, IL, USA). Differences were considered significant at *p*<0.05.

## Results

Of the 1,978 women who visited the Urogynaecology Clinic during the study period, 621 women were sexually active and bothered by urinary incontinence. While 537 women were willing to join the interview, 505 women completed the quality of life assessments and urodynamic measurements. A total of 281 (56%) women answered affirmatively having experienced coital incontinence, and 224 (44%) did not. Only 25 (9%) patients voluntarily reported this condition. Based on the demographic characteristics of patients with and those without coital incontinence (Table 1), women with coital incontinence seemed to be multiparous (*p* = 0.042), had higher body mass index (*p* = 0.027), fewer normal urodynamics (*p* = 0.041) and lower maximal urethral closure pressure (≤ 30 cmH2O) (*p* = 0.001). While 10% (29/281) had mixed incontinence, and 5% (15/281) had detrusor-overactivity, 64% (181/281) of the patients had urodynamic stress incontinence, showing coital incontinence was prevalent in women with stress incontinence (Table 1). There was no significant difference regarding the types of incontinence between women with and without coital incontinence.

**Table 1:** Demographic and clinical characteristics of women with and without coital incontinence.

	With coital incontinence (n = 281)	Without coital incontinence (n = 224)	*p*
Age(range), yr	52.1 ± 8.3 (29–74)	50.6 ± 9.5 (25–80)	0.061
Parity, n	2.9 ± 1.3	2.6 ± 1.3	0.042
Body mass index, kg/m^2^	24.7 ± 3.4	23.3 ± 3.5	0.027
Menopause, n	126 (45%)	90 (40%)	0.574
Previous hysterectomy, n	34 (12%)	17 (8%)	0.243
Hormone therapy, n	23 (8%)	9 (4%)	0.077
Urodynamic assessments, n			
USI	181 (64%)	125 (56%)	0.190
MI	29 (10%)	18 (8%)	
DO	15 (5%)	8 (4%)	
Normal urodynamic assessments, n	56 (20%)	73 (33%)	0.041
Maximal detrusor muscle contraction pressure in women with DO, cmH2O	55.2 ± 27.8	58.6 ± 31.8	0.254
Maximal urethral closure pressure, n			
20 cmH2O	4 (1%)	1 (0.4%)	0.387
≤ 30 cmH2O	34 (12%)	5 (2%)	0.001
≤ 40 cmH2O	53 (19%)	34 (15%)	0.283

Data are presented as mean ± standard deviation or as number of patients.

MI, mixed incontinence; USI, urodynamic stress incontinence; DO, detrusor over-activity.

Women with mixed incontinence had the worst sexual quality of life (*p* = 0.001) and incontinence-related symptom distress (*p* = 0.014) (Table 2).

**Table 2:** Quality of life with regards to different types of incontinence in women with coital incontinence.

	USI (n = 181)	MI (n = 29)	DO (n = 15)	*p*
UDI-6	8[4–13]	11[5–18]	7[5–14]	0.014
IIQ-7	9[5–16]	10[4–18]	9[5–16]	0.347
PISQ-12	26[12–35]	22[9–33]	30[19–38]	0.001

Data are presented as median [interquartile range].

MI, mixed incontinence; USI, urodynamic stress incontinence; DO, detrusor over-activity; UDI-6, short form of Urogenital Distress Inventory; IIQ-7, short form of Incontinence Impact Questionnaire; PISQ-12, short form of Pelvic Organ Prolapse/Urinary Incontinence Sexual Questionnaire.

For the frequency of coital incontinence, 50% (84/281) of the women reported rarely or sometimes having incontinence during coitus, 33% (92/281) often, and 17% (48/281) always, indicating half of the women had coital incontinence more frequently than sometimes. There was no significantly different frequency regarding the types of incontinence (*p* = 0.153) (Table 3).

**Table 3:** Frequency of coital incontinence with regards to different types of incontinence.

Frequency of coital incontinence	USI (n = 181)	MI (n = 29)	DO (n = 15)	*p*
Sometimes or rarely	73 (40%)	7 (24%)	8 (53%)	0.153
Often	70 (39%)	15 (52%)	4 (27%)	
Always	38 (21%)	7 (24%)	3 (20%)	

MI, mixed incontinence; USI, urodynamic stress incontinence; DO, detrusor over-activity.

According to univariate analysis, higher body mass index (OR 2.47, *p* = 0.027) and lower maximal urethral closure pressure (≤ 30 cmH2O) (OR 4.56, *p* = 0.007) were the possible indicators of coital incontinence. After multivariate logistic regression analysis was conducted, maximal urethral closure pressure ≤ 30 cmH2O was an independent risk factor for coital incontinence was identified (OR 3.93, *p* = 0.042) (Table 4).

**Table 4:** Analysis of risk factors for coital incontinence.

Patient characteristics	Univariate analysis		Multivariate analysis	
	OR (95% CI)	*p*	OR (95% CI)	*p*
Age, yr				
< 60	0.87 (0.49–4.79)	0.324		
≥ 60	1.99 (0.81–5.01)	0.525		
Multiparous, n (Reference: nulliparous)	1.39 (0.92–3.77)	0.241		
Body mass index ≥ 25 kg/m^2^ (Reference: BMI < 25)	2.47 (1.13–4.32)	0.027	0.93 (0.71–3.34)	0.317
Menopause, n (Reference: pre-menopause)	0.99 (0.94–1.08)	0.293		
Hormone therapy, n (Reference: none)	1.29 (0.87–3.13)	0.455		
Previous hysterectomy, n (Reference: none)	1.46 (0.78–2.71)	0.249		
Maximal detrusor muscle contraction pressure, cmH2O (Per 1 unit increase)	1.57 (0.93–2.73)	0.143		
Maximal urethral closure pressure ≤ 30 cmH2O (Reference: MUCP > 30)	4.56 (1.51–13.79)	0.007	3.93 (1.21–40.44)	0.042

OR, odds ratio; BMI, body mass index; MUCP, maximal urethral closure pressure.

## Discussion

The present study revealed that very few patients voluntarily consulted on coital incontinence, although it was not an uncommon symptom in women with urinary incontinence. Consistent with the clinical observation of El-Azab at al. that coital incontinence was negatively correlated with abdominal leak point pressure (urethral competence) [3], we also noted maximal urethral closure pressure ≤ 30 cmH2O associated with coital incontinence, indicating that urethral function plays an important role in maintaining continence during coitus. El-Azab et al. tried to determine the indicators for coital incontinence by assessing urodynamic measurements and anatomic anomalies using magnetic resonance imaging [3]. Similarly, they noted the majority of the patients (89%) had stress incontinence. Coital incontinence was correlated with the severity of stress incontinence and urethral incompetence. There was no specific anatomical anomaly discovered by magnetic resonance imaging. They thus concluded that coital incontinence is almost invariably a symptom of stress incontinence with urethral sphincter incompetence [3].

Similar result was also reported by studies of Madhu et al. [5] and Pastor [14]. Madhu et al. conducted a retrospectively study to analyse 1391 patients who had coital incontinence and underwent urodynamic examination from 1991 to 2009. They noted urodynamic-proven stress incontinence was significantly associated with coital incontinence [5]. Pastor conducted a review about women expelled fluids during sexual arousal and at orgasm. He also proposed that coital incontinence was a pathological sign caused by urethral disorder [14]. Moreover, El-Azab at al. reported there was no different amplitude of detrusor contraction pressure between detrusor overactivity women with and without coital incontinence. They speculated coital incontinence at orgasm not responding well to anticholinergics was due to urethral incompetence rather than severe refractory from of detrusor overactivity [3]. According to our data, we also noted the amplitude of detrusor contraction pressure was not different between women with and without coital incontinence. However, due to the limited numbers of detrusor overactivity subjects and unknown the timing when coital incontinence did occur (at penetration or orgasm), there is insufficient evidence to explain the pathophysiology of coital incontinence in detrusor overactivity subjects. Coital incontinence at orgasm in women with detrusor overactivity may be associated with a more complex pathophysiologic mechanism that combines neural transduction, urethral function, and detrusor activity.

This study showed the incidence of coital incontinence was up to 56% and prevalent in women with stress incontinence. The possible reasons to explain the high incidence and different clinical observations from the review by Serati et al. that the incidence ranged 10–27% [2] may be due to the different methodologies, and the frequency of coital incontinence. Patients who go to a clinic theoretically have more severe symptoms, are willing to consult on this embarrassing symptom, and to receive urodynamic measurements. However, stress incontinence is the most common clinical symptom and the indication for urodynamics [4]. Given the nature of this condition, it is no wonder that the majority of these patients had stress incontinence. This study evaluated the frequency of coital incontinence among women with incontinence. Based on our data, half of the patients reported coital incontinence rarely or sometimes, 33% (92/281) often, and 17% (48/281) always. For patients that reported rarely or only sometimes experiencing such symptoms, they may not be willing to discuss this with physicians because it is not a “frequent” problem. If not counting these patients, the prevalence of coital incontinence was 28% (140/505) and that was similar with the data of previously published studies.

Another interesting finding related to our data was that it revealed women with coital incontinence were more prone to have abnormal urodynamic diagnosis which echoed the findings of Jha et al. who reported normal urodynamics were less likely in women with coital incontinence [4]. This may indicate that coital incontinence is a specific symptom suggesting abnormal urodynamic findings and deteriorated urethral function. Some studies have reviewed the influence of different types of incontinence on female sexual function, and showed the conflicting data [15,16]. Using a valid questionnaire, Urwitz-Lane et al. reported that sexual function was not altered in different types of incontinence [15]. In contrast, Coksuer et al. reported that stress incontinence affected sexual function more than detrusor overactivity [16]. In the present study, the frequency of coital incontinence was not significantly different regarding the type of incontinence; however, patients with mixed incontinence had the worst sexual function and quality of life. Theoretically, stress incontinence and detrusor overactivity are from different pathophysiologic processes. Stress incontinence is associated with bladder neck hyper-mobility and urethral incompetence, while detrusor over-activity is associated with detrusor muscle instability [4]. Since mixed incontinence is a combination of both, this may explain why such patients have the worst sexual function.

This study has a number of limitations that should be noted. Data on when coital incontinence occurred, either during penetration or at orgasm, was not obtained. Not performing a semi-structured interview was also a limitation. The predominance of stress incontinence patients may also cause some bias to the analysis of risk factors. The treatment outcomes of coital incontinence by either medication or surgery were not followed up. That was due to this was an observational study, and some of the women recruited from clinic were not willing to further treatment. As a result, the choice of treatment remains unclear. The merit of this study is to evaluate the frequency of coital incontinence and quality of life in a large sample size with valid questionnaires and urodynamic measurements.

## Conclusions

The prevalence of coital intercourse in urinary incontinence women was high. Coital incontinence in these women was associated with abnormal urodynamic diagnosis and urethral dysfunction.
